# Perception of injury risk among amateur Muay Thai fighters

**DOI:** 10.1186/s40621-016-0099-y

**Published:** 2017-01-16

**Authors:** Stephen Strotmeyer, Reidar P. Lystad

**Affiliations:** 1 Australian Institute of Health Innovation, Faculty of Medicine and Health Sciences, Macquarie University, Sydney, Australia; 2 Neuroscience Research Australia (NeuRA), Randwick, 2031 NSW Australia

**Keywords:** Perceived risk, Perceived comparative risk, Comparative optimism, Unrealistic optimism, Athletic injuries

## Abstract

**Background:**

Muay Thai is a style of kickboxing that allows full-contact blows to an unprotected head, torso and legs, and, as in any combat sport, there is an inherent risk of injury. Previous observational studies have shown there is a substantial risk of injury in competitive kickboxing. None of these studies, however, have investigated the potential role of psychological risk factors and, consequently, little is known about the perception of injury risk among these athletes. Notwithstanding the important role risk perception may play in the occurrence and prevention of sports injuries, there is very limited empirical data pertaining to athletes in full-contact combat sports such as Muay Thai.

Because the development and successful implementation of effective injury prevention policies for combat sports are likely to benefit from an increased understanding of the perception of injury risk and sport safety attitudes and behavior of its participants, further study is warranted.

**Methods:**

Muay Thai fighters were invited to complete an online survey in which they rated the perceived risk of injury in a range of different sports, including Muay Thai kickboxing. Perceived comparative risk was obtained indirectly by subtracting perceived risk of injury to oneself from perceived risk of injury to a peer. Data were analyzed using descriptive statistics, comparison of means, and ordinal logistic regression.

**Results:**

Contrary to the best available epidemiological evidence, Muay Thai fighters perceived the risk of injury in their own sport to be average and significantly lower than that in other collision and contact sports, including popular combat sports such as boxing and mixed martial arts. On average, Muay Thai fighters perceived their own risk injury to be significantly lower compared to their peers (*p* < 0.001).

**Conclusions:**

There appears to be a mismatch between injury risk perception and actual risk among Muay Thai fighters. Moreover, these athletes also exhibit a slight degree comparative optimism or unrealistic optimism. Because behavior is determined by perceived rather than actual risk, underestimation of injury risk and concomitant overestimation of ability to negotiate risk may lead to an increased frequency of injury. Future injury prevention strategies in combat sports such as Muay Thai kickboxing should consider educational- and psychosocial-based interventions that include efforts to correct erroneous beliefs and attitudes about actual risk of injury in the sport.

## Background

Although regular physical activity is widely encouraged for its health benefits, participation in sport is not without risk. Indeed, sports injury is identified as a major public health problem in Western societies. Based on stress theory adapted from earlier theoretical models, (Andersen and Williams 1988; Williams and Andersen 1998) Junge (Junge 2000) proposed an integrative theoretical model of the influence of psychological factors on injury occurrence. Investigations to date have focused on psychological factors associated with the susceptibility to injury during sports participation, namely: psychosocial stressors (e.g., life events and everyday problems), coping resources (e.g., coping skills, social support and health behavior) and personality traits (e.g., general anxiety, competitive anxiety, and mental fatigue).(Junge 2000).

There are, moreover, good reasons to think that athletes’ subjective perception of risk of injury is likely to influence their sports safety behavior. For instance, cognitive-behavioral theories propose that athletes’ decisions are based on assessing the probability of outcomes and consequences of engaging, or not engaging, in a behaviour (Ajzen 1991; Janz and Becker 1984; Siesmaa et al. 2011). This suggests that athletes who underestimate the risk of injury engage in more risk-taking behaviors, whereas athletes who overestimate the risk adopt preventive behaviors. Indeed, studies have indicated that perceived risk is a good indicator of actual risk-taking behaviour (Kontos 2004).

Several factors might influence the relationship between perceived risk and risk-taking, thereby making it difficult to ascertain the true nature of the relationship (Kontos 2004; Morrongiello and Lasenby‐Lessard 2007). These factors include: individual characteristics such as previous experiences (both positive and negative), personal motivations, gender, age, and behavioral attributes; family factors such as parenting style, parenting attributes, and sibling effects; and social-situational factors such as observational influences, situational influences, and persuasion influences (Morrongiello and Lasenby‐Lessard 2007).

One particular individual characteristic is athletes’ perceived confidence to negotiate their own risk of injury. Bandura (Bandura 1997) theorized that individuals with high self-efficacy—that is, those who believe they can succeed in specific situations—may overestimate their own ability, which subsequently may lead to underestimating their actual risk of injury and thus deciding to engage in riskier behaviour (Llewellyn et al. 2008). Conversely, individuals with low self-efficacy may underestimate their own ability to negotiate risk, which in turn results in overestimating the risk of injury and adopting less risky behavior (i.e., “playing it safe”).

There is a substantial body of health psychology literature describing people’s perception of risk compared to that of their peers (Klein and Helweg-Larsen 2002). Some individuals have a tendency to report being less likely than their peers to experience negative events (e.g., injury) and more likely than others to experience positive events (e.g., winning a tournament); whereas other individuals have a tendency towards the converse, that is, to report being more likely than others to experience a negative event and less likely to experience a positive event. The former tendency is known as *comparative optimism* or unrealistic optimism, while the latter is referred to as *comparative pessimism* or unrealistic (Klein and Helweg-Larsen 2002; Martha and Laurendeau 2010; Moen and Rundmo 2005).

Although such *perceived comparative risk* (PCR) may influence athletes’ decisions to engage in risk-taking behavior, and thus also be linked the occurrence of injury, only a few studies have investigated PCR in high-risk athletic populations to date (Martha and Laurendeau 2010; Moen and Rundmo 2005; Lystad et al. 2015). Moreover, the findings have been contradictory in that high-risk athletes have reported their risk of injury to be either similar to that of their peers, (Martha and Laurendeau 2010) or comparatively optimistic (Moen and Rundmo 2005; Lystad et al. 2015).

Muay Thai is a style of kickboxing that allows full-contact blows to an unprotected head, torso and legs, and, as in any combat sport, there is an inherent risk of injury. Previous observational studies have shown there is a substantial risk of injury in competitive kickboxing (Lystad 2015a; Zazryn et al. 2003). None of these studies, however, have investigated the potential role of psychological risk factors and, consequently, little is known about the perception of injury risk among these athletes. Notwithstanding the important role risk perception may play in the occurrence and prevention of sports injuries, there is very limited empirical data pertaining to athletes in full-contact combat sports such as Muay Thai. Because the development and successful implementation of effective injury prevention policies for combat sports are likely to benefit from an increased understanding of the perception of injury risk and sport safety attitudes and behavior of its participants, further study is warranted (Finch et al. 2002).

The purpose of this study, therefore, was to examine the injury risk perception among Muay Thai fighters. The specific objectives were: (1) to determine their perceived risk of injury in a range of sports, including Muay Thai; (2) to determine their perceived risk of injury to themselves; (3) to determine their perceived comparative risk of injury; and (4) to explore factors which may predict their perceived risk of injury and perceived comparative risk of injury.

## Methods

Three-hundred eighty-seven Muay Thai fighters based in the United States, aged 18 years or over, competing at the largest national amateur tournament were eligible to participate in the study. Invitations to participate were forwarded via an electronic mailing list, and eligible participants were asked to complete an anonymous online survey administered via Qualtrics Research Suite software (Qualtrics, Provo, UT, USA). One-hundred seventy-five fighters completed the survey for a 42.5% response rate. The study was approved by The University of Pittsburgh Institutional Review Board.

The survey instrument was based on a survey previously developed and validated by Siesmaa and colleagues (Siesmaa et al. 2011). It included questions concerning demographic information (e.g., age, gender, and ancestry), and safety and injury risk in sport. Slight modifications to the original generic survey were introduced to directly address a population of Muay Thai fighters, namely: (1) phrases like “the sport you play most” were substituted with “Muay Thai”; (2) the list of sports the respondents were asked to rate was altered to include specific combat sports (i.e., boxing, mixed martial arts, judo, and karate) and popular American sports (i.e., American football and baseball), at the expense of a few other sports (i.e., athletics, Australian Rules Football, cricket, gymnastics, netball, skateboarding, roller skating/blading, and trampolining); and (3) in an attempt to mitigate the effect of scale attenuation, (Harris and Hahn 2011; Otten and van der Pligt 1996) the perceived risk of injury was measured using a 5-point Likert scale ranging from −2 (very low chance of getting injured) to 2 (very high chance of getting injured) instead of a 3-point Likert scale. This is also more in line with other studies of perceived comparative risk, which have utilized either 5-point (Lystad et al. 2015; Deroche et al. 2012; Rutter et al. 1998) or 7-point (Martha and Laurendeau 2010; Moen and Rundmo 2005) Likert scales.

Participants were asked to rate the risk of injury to an equal referent (i.e., an average athlete of the same age, gender, and level of experience as themselves) for a range of sports with varying degrees of contact, including taekwondo (PR_OTHER_). Sports were classified as *collision*, *contact*, *limited-contact* or, *non-contact* in accordance with a recent policy statement from the American Academy of Pediatrics (Rice 2008). In brief, the classification pertains to whether athletes purposely hit or collide with each other or inanimate objects (including the ground) with great force (*collision*), routinely with less force (*contact*), infrequently or inadvertently (*limited-contact*), or never (*noncontact*). The respondents were also asked to rate their own risk of injury in Muay Thai (PR_SELF_). Perceived comparative risk was obtained indirectly by subtracting PR_SELF_ from PR_OTHER_ for each respondent, and categorized as follows: positive scores (ranging from +1 to +4; i.e., PR_SELF_ < PR_OTHER_) indicate comparative optimism; zero scores indicate neutrality (PR_SELF_ = PR_OTHER)_; and negative scores (ranging from −1 to −4; i.e., PR_SELF_ > PR_OTHER_) indicate comparative pessimism. This method of subtracting a single item from another to measure perceived comparative risk has been successfully utilized in previous studies (Martha and Laurendeau 2010; Lystad et al. 2015; Deroche et al. 2012).

Prior to analysis, all survey data were exported to an electronic spreadsheet for further data cleaning and coding. Descriptive statistics were used to describe demographic information and responses to questions and statements, including risk perception (PR_SELF_, PR_OTHER_, and PCR). That is, frequencies and proportions were reported for categorical variables, while means and standard deviations or 95% confidence intervals (CI) were reported for continuous variables. Paired student t-tests were used to determine if there were any significant differences between the respondents’ perceived risk of injury in taekwondo and their perceived risk of injury in other sports. Ordinal logistic regression models were fitted to determine if there were and relationships between risk perception (PR_SELF_, PR_OTHER_, or PCR) and demographic variables. All odds ratios derived from ordinal logistic regression models were reported with both 95% CI and *p*-values. The models were checked using tests of non-proportional odds for all variables. All statistical analyses were performed using R, version 3.3.1 (R Core Team, Vienna, Austria).

## Results

One-hundred and seventy-five Muay Thai fighters completed the online survey, of which 61 (35%) were female. The mean age of the respondents was 30.0 ± 8.0 (range: 18 to 55) years. The frequencies and proportions of respondents by self-reported ancestry were as follows: 111 (65%) European, 16 (9%) Asian, 15 (9%) Hispanic, 12 (7%) African, and 18 (10%) ‘other’. Overall, the respondents were relatively novice fighters, with 126 (77%) being categorized as inexperienced and 38 (23%) as experienced. Only 67 (40%) of the respondents reported having participated in a Muay Thai fight within the preceding 6 months.

In regard to the statement ‘Muay Thai is safer than most other sports’, the frequencies and proportions of responses were as follows: 34 (20%) agreed, 74 (33%) disagreed, 55 (44%) were neutral, and 4 (2%) did not know. In regard to the statement ‘The protective gear in Muay Thai is adequate to prevent injuries’, the frequencies and proportions of responses were as follows: 90 (54%) agreed, 37 (22%) disagreed, 33 (20%) were neutral, and 7 (4%) did not know.

Table 1 provides an overview of the frequency and proportion of respondents’ perceived risk of injury for an equal referent participating in a range of sports, the mean rating of risk with 95% confidence interval, and comparisons of mean rating of risk in Muay Thai versus (referent) versus other sports using paired t tests. As expected, the respondents perceived the risk of injury in collision sports to be greater than in contact sports, which in turn was greater than in limited-contact and non-contact sports. The respondents rated an equal referent’s chance of getting injured while participating in a Muay Thai fight to be slightly above average (PR_OTHER_ 0.05 [95% CI: −0.07, 0.17]). The risk of injury in Muay Thai was rated significantly lower than for most other collision sports (i.e., rugby, American football, mixed martial arts, and boxing); significantly greater than for one collision sport (i.e., karate), one contact sport (i.e., basketball), and all limited contact-sports (i.e., baseball and cycling) and non-contact sports (i.e., swimming).

**Table 1:** Frequency and proportion of respondents’ perceived risk of injury for an equal referent participating in a range of sports, mean rating of risk with 95% confidence interval (CI), and comparison of mean rating of risk in Muay Thai versus (referent) versus other sports using paired t tests

	Chance of getting injured						
Sport	Very low	Low	Average	High	Very high	Mean [95%CI]	*p* value
Collision							
Rugby	-	5 (3%)	38 (23%)	74 (45%)	49 (30%)	1.01 [0.88, 1.13]	< .001
American football	-	3 (2%)	46 (28%)	69 (41%)	49 (29%)	0.98 [0.86, 1.10]	< .001
Mixed martial arts	-	8 (5%)	49 (30%)	74 (45%)	35 (21%)	0.82 [0.69, 0.94]	< .001
Boxing	1 (1%)	20 (12%)	80 (48%)	50 (30%)	16 (10%)	0.36 [0.23, 0.49]	< .001
Judo	3 (2%)	29 (17%)	92 (55%)	35 (21%)	8 (5%)	0.10 [−0.03, 0.22]	.478
Muay Thai	2 (1%)	32 (19%)	97 (58%)	28 (17%)	8 (5%)	0.05 [−0.07, 0.17]	referent
Karate	10 (6%)	53 (32%)	85 (51%)	16 (10%)	2 (1%)	−0.32 [−0.44, −0.20]	< .001
Contact							
Soccer	11 (7%)	44 (26%)	78 (47%)	26 (16%)	8 (5%)	−0.14 [−0.29, −0.00]	.043
Basketball	7 (4%)	62 (37%)	75 (45%)	21 (13%)	1 (1%)	−0.32 [−0.44, −0.20]	< .001
Limited-contact							
Baseball	26 (16%)	83 (50%)	48 (29%)	9 (5%)	1 (1%)	−0.74 [−0.87, −0.62]	< .001
Cycling	39 (23%)	77 (46%)	40 (24%)	10 (6%)	-	−0.87 [−1.00, −0.74]	< .001
Non-contact							
Swimming	89 (53%)	66 (40%)	11 (7%)	1 (1%)	-	−1.46 [−1.55, −1.36]	< .001

The respondents perceived their own chance of getting injured while competing in Muay Thai kickboxing to be slightly below average (PR_SELF_ -0.12 [95% CI: −0.23, 0.00]), which was significantly lower than their rating for an equal referent (mean difference 0.17, t_166_ = 3.625, *p* < 0.001). In regard to perceived comparative risk, the frequency and proportion of respondents by category were as follows: 33 (20%) comparatively optimistic, 124 (74%) neutral, and 10 (6%) comparatively pessimistic.

Ordinal logistic regression analyses were undertaken to explore the relationships between perceived risk to oneself (PR_SELF_), perceived risk of injury to an equal referent (PR_OTHER_), or perceived comparative risk (PCR) and potential demographic predictor variables. The models did not violate the proportional odds assumption. As Table 2 shows, no significant relationships were detected between demographic variables and PR_SELF_, PR_OTHER_, or PCR.

**Table 2:** Ordinal logistic regression models exploring the relationships between demographic variables and perceived risk to oneself (PR_SELF_), perceived risk of injury to an equal referent (PR_OTHER_), or perceived comparative risk (PCR)

Variable	Model: PR_SELF_		Model: PR_OTHER_		Model: PCR	
	OR [95% CI]	*p* value	OR [95% CI]	*p* value	OR [95% CI]	*p* value
Age in years	1.01 (0.97, 1.05)	.693	1.03 (0.99, 1.08)	.105	1.04 [1.00, 1.10]	.079
Female (ref. male)	1.57 (0.80, 3.12)	.190	1.85 (0.96, 3.59)	.066	1.26 [0.58, 2.72]	.565
Experienced fighter (ref. no)	0.89 (0.38, 2.08)	.781	0.82 (0.36, 1.88)	.645	1.13 [0.43, 2.89]	.802
Recent fight (ref. no)	0.62 (0.30, 1.25)	.183	0.77 (0.39, 1.53)	.465	1.14 [0.51, 2.52]	.750
Ancestry (ref. European)						
Hispanic	0.93 (0.30, 2.94)	.904	0.97 (0.30, 3.05)	.953	0.63 [0.16, 2.35]	.509
African	1.47 (0.42, 5.29)	.554	2.90 (0.83, 10.06)	.093	2.26 [0.53, 8.82]	.251
Asian	2.57 (0.87, 7.69)	.090	1.44 (0.50, 4.13)	.450	0.33 [0.09, 1.25]	.107
Other	0.77 (0.26, 2.36)	.646	1.17 (0.41, 3.37)	.774	1.91 [0.57, 5.96]	.276

*CI* confidence interval, *OR* odds ratio, *PCR* perceived comparative risk, *PR*_*SELF*_ perceived risk of injury to oneself, *PR*_*OTHER*_ perceived risk of injury to an equal referent

## Discussion

This is the first study to examine the perception of injury risk in Muay Thai kickboxing. It reveals that Muay Thai fighters perceive the risk of injury in their own sport to be slightly below average risk, which is significantly less than their perceived risk of injury for other collision sports, including popular combat sports such as boxing, mixed martial arts, and judo. Although Muay Thai fighters, on average, exhibit slight comparative optimism, the majority of fighters believe that their own ability to negotiate risk is similar to the ability of their peers.

The injury incidence rate per 1000 athlete-exposures in kickboxing has been reported to range between 109.7 and 155.4, (Lystad 2015b) while in other full-contact combat sports it has been reported to be as follows: 41.2–115.1 in judo, (Lystad 2015b) 45.2–214.3 in karate, (Lystad 2015b) 77.7–250.6 in boxing, (Lystad 2015b) and 228.7 in mixed martial arts (Lystad et al. 2014). In light of the available literature, these findings suggest that Muay Thai fighters underestimate the actual risk of injury in their own sport relative to other collision and contact sports. This apparent underestimation of injury risk is likely due to the effects of the respondents’ voluntary exposure, familiarity, and personal experience with Muay Thai, all of which are factors known to attenuate perceived risk of injury (Morrongiello and Lasenby‐Lessard 2007). In particular past successful experiences with risk taking (i.e., avoiding injury or mastering a new skill) in Muay Thai kickboxing would be very likely to lead to increased understanding and ability to control hazard.

In regard to perceived comparative risk, the Muay Thai fighters in this study appeared to believe that their own ability to negotiate risk was similar to the ability of their peers (i.e., PR_SELF_ was rated similar to PR_OTHER_). This finding corresponds with previous studies reporting that taekwondo athletes do not express any significant degree of comparative optimism (Lystad et al. 2015). There is, however, a sizeable minority (20%) of Muay Thai fighters who exhibit comparative optimism, and it remains unknown whether these athletes are more injury prone compared to their peers who are not comparatively optimistic. Hence, further research is needed to elucidate the role of perceived comparative risk in the risk-taking decision-making process in high-risk athletes.

### Limitations

The main limitations of this study pertain to the fact that participants selected themselves into the study. This may have resulted in a sample whose opinions and beliefs were not necessarily representative of the whole population of Muay Thai fighters. In addition, the generalizability of the findings herein may be limited due to the sampling being restricted to amateur Muay Thai fighters in the United States of America, which may, for reasons unknown, perceive the risk of injury differently to Muay Thai fighters in other parts of the world. Further, the amateur sample was required to wear protective equipment (headgear, shin pads, gloves and elbow pads). A professional fighter who would not wear any protection other than gloves may report different levels of risk given that they are less protected and also the bout duration would be considerably longer in round time and the number of rounds fought. Additionally, Muay Thai practitioners, or those who train but do not compete may report vastly different perceptions given varying levels of participation and contact. Future studies investigating the intensity and duration of training exposures, and the practitioner level, especially comparing amateur to professional fighters would be a great addition in future studies. The survey instrument used was based on that developed and validated by Siesmaa and colleagues,(Siesmaa et al. 2011) with minor modification to make the generic survey into a Muay Thai-specific survey. While we believe that the validity holds with this modification, we did not have the resources to re-validate the modified survey. Lastly, attenuated response scales such as the 5-point Likert scale can be problematic as they may be subject to floor and ceiling effects (Harris and Hahn 2011). As Otten and van der Pligt (Otten and van der Pligt 1996) observed, participants may report greater optimism when they are given an attenuated scale than when they are given a continuous scale, thus making it difficult to interpret the findings. Future studies should consider adopting a continuous scale as recommended by Harris and Hahn (Harris and Hahn 2011).

## Conclusion

Muay Thai fighters appear to underestimate the risk of injury in their own sport relative to other collision and contact sports, including popular combat sports such as boxing, mixed martial arts, and judo. These fighters, moreover, seem to believe that their own ability to negotiate risk is greater than the ability of their peers. Because behavior is determined by perceived rather than actual risk, overestimation of ability to negotiate risk and concomitant underestimation of risk may lead to an increased frequency of injury. Future injury prevention strategies in combat sports such as Muay Thai kickboxing should consider educational- and psychosocial-based interventions that include efforts to correct erroneous beliefs and attitudes about actual risk of injury in the sport.

### Practical implications

- There appears to be a mismatch between injury risk perception and actual risk among Muay Thai fighters.
- Because behavior is determined by perceived rather than actual risk, underestimation of injury risk and concomitant overestimation of ability to negotiate risk may lead to an increased frequency of injury
- Future injury prevention strategies in combat sports should consider educational- and psychosocial-based interventions that include efforts to correct erroneous beliefs and attitudes about actual risk of injury in the sport.
